# Ableism-sensitive, self-reflective emotion work as part of inclusive teacher education

**DOI:** 10.3389/fsoc.2025.1401775

**Published:** 2025-02-06

**Authors:** Mandy Hauser

**Affiliations:** ^1^Leipzig University, Leipzig, Germany; ^2^Department of Inclusive Education and Participation in the Context of Intellectual Disabilities, Institute for Special Needs and Inclusive Education, Faculty of Education, Leipzig University, Leipzig, Germany

**Keywords:** emotion work, inclusive teacher education, unlearning ableism, sociology of emotions, group process, reflexivity

## Abstract

In this perspective article, emotions are considered as an inherent component of ableist practices, and the question is explored of how ableism-sensitive, self-reflective emotion work can be designed for inclusive teacher education. In this process, connections to the Sociology of Emotions are established, with particular emphasis on the collectivity and sociality of emotions. Within this context, self-reflective emotion work is integrated into the concept of “unlearning ableism” and argued for its implementation as a systemically oriented group process. Finally, questions regarding the design of emotion work and its implementation in a manner critical of ableism are discussed.

## 1 Introduction

Emotions are an inherent part of ableist practices (Wechuli, 2022) and manifest in various forms across all levels of educational relationship and interactions within the school context, significantly influencing teaching and learning processes (Zhongling et al., 2022). For this reason, engaging with (one’s own) emotionality is also significant for inclusive teacher education. Inclusion as a key concept in Disability Studies refers to the equal access, participation, and involvement of all individuals in all socially relevant domains. The pedagogical practice of segregation maintains separating structures of thought and action and it reproduces ableism as an order of difference characterized by the valuation and devaluation of individuals in relation to (dis)ability expectations and attributions. The concept of ableism was initially developed within the disability movement and further elaborated in Disability Studies. The segregating education system and the teachers acting within it are identified as central to ableist subject production, while inclusive pedagogy is conceptualized as its counter-strategy (Buchner, 2022). Embedded in this is the demand on teacher education for inclusion to critically reflect upon the often deeply sociocultural and biographically rooted and internalized “expectations of abilities and ableist assumptions” (Buchner, 2022) and associated emotions and emotional patterns. Therefore this perspective article aims to explore how self-reflective emotion work can be designed within inclusive teacher education. For this end, references to the Sociology of Emotions will first be outlined, followed by a description of self-reflective emotion work as part of a process of unlearning ableist ways of thinking, feeling and acting. This will involve raising potential perspectives and questions regarding the implementation of self-reflective practices in teacher education.

## 2 Fundamental ideas from the Sociology of Emotions

Fundamental to my discussion are conceptual frameworks that guide the Sociology of Emotions, with three aspects of particular relevance.

The sociological perspective on emotions implies that emotions carry cultural significance and manifest their expression in the formation of social relationships and emotions “are shaped, and in fact constructed, by social conditions” (Holmes, 2010, p. 144). This means that social arrangements are inherently emotional arrangements (Illouz, 2004), and social practice is invariably emotional practice. According to Neckel (2006), emotions represent the most immediate manifestation of the “social perception of societal conflicts about power and morality” (Neckel, 2006, p. 133, author’s translation). This is because emotional responses to violations of moral norms and the associated normative expectations occur spontaneously, and they provide clues about their presence as well as their structure and order. Thus, emotions represent fundamental normative dimensions of meaning within the cultural practices of social groups and are a reflection of social conditions and inequalities. This aspect can be linked to one of the main concerns of Disability Studies: to investigate “how society and culture shape the way we react to dis/ability and what this tells us about underlying norms” (Wechuli, 2022, p. 143).
Building upon this approach, emotions must be conceptualized as highly complex, context-dependent phenomena (Ahmed, 2004). This implies that emotions are not confined to the individual level of the perceiving subject but are deeply interwoven with ableist structures and cultures as collective emotions and they are far from being “merely reflexes of social positions, outcomes of physiological stimuli, and subjective correlates of role expectations” (Neckel, 2006, p. 134, author’s translation). According to Ahmed (2004), emotions in this sense are to be understood as relational, and the subject’s sensations are influenced both by the internal context, such as past subjective experiences and interpretations, and the external context, such as collective history or structures. In doing so, Ahmed breaks with “foundational distinctions in Western philosophy between reason and feeling as well as between intellect and emotion” (Ural, 2023, p. 34, author’s translation). Furthermore, Ahmed’s perspective on emotions as responsive is significant. Emotions are not purely subjective and individualistic. Rather, the emotions subjectively experienced are socially mediated and are in contact with emotions that circulate in a particular social and culturally influenced manner: “They move and they are not just social in the sense of mediated, but they actually show how the subject arrives into a world that already has affects and feelings circulating in very particular ways” (Schmitz and Ahmed, 2020, p. 98).
This assumption is accompanied by the idea that emotions are not limited to affective, unconscious states but also encompass reflexive-cognitive components as well as motivational and action-related aspects. Emotion and cognition are in an interdependent relationship and following this perspective, it becomes possible to access one’s own emotions and engage in reflective processing of them.

## 3 Self-reflective emotion work within the context of “unlearning ableism”

For the self-reflective work on one’s own emotions, the term “emotion work” can be used, tracing back to the works of Hochschild (1983). The term refers to processes of emotion regulation, involving the production and display of desired emotions while suppressing undesired emotions and emotional states (Werner, 2016). Hochschild summarizes the processes as “the management of feeling to create a publicly observable facial and bodily display” (Hochschild, 1983; Werner, 2016, n.p., author’s translation) captures them as “intentional generation, influence, representation, and regulation of one’s and others emotional states” and concretized: “Who, when, in which situations what one feels, and how the individual expresses these emotions to others constitute a socially determined and power-permeated, complex process.” So-called “feeling rules” define the norms of emotional behavior in various situations and provide a valuable approach to understanding emotions as social phenomena (Holmes, 2010).

Below, I draw upon the ideas of Hochschild (1983) and Werner (2016), connecting them with the notion of self-reflective work with and on one’s own emotions. I aim to specify approaches and meanings of emotion work for the professionalization of teachers for inclusion. In this regard, it involves empowering prospective teachers to become aware of unconscious, prereflective emotional aspects, to resist feeling rules, and to acknowledge all facets of emotions independently of social evaluation. This entails allowing oneself to experience emotions and influencing emotions through reflexive engagement. This process of recognizing and influencing individual and collective (ableist) emotional patterns can be considered as part of a persistent and intensive process termed “unlearning ableism” as described by Buchner (2022) and used by Disability Studies to question and transform ableist practices and policies (Danforth and Gabel, 2016).

While this process can be initiated during teachers’ training, it should never be regarded as complete due to its complexity and socio-cultural conditioning. Unlearning is like learning an essential part of educational processes and, according to Spivak (1996), contributes significantly to the repoliticization of pedagogy. Spivak (1996) coined the concept of unlearning as part of postcolonial theory, with reference to epistemic violence, and understands it from a deconstructive perspective. It involves recognizing the “interweaving of learning and education with power and domination” (Castro Varela, 2017, n.p., author’s translation) and developing an awareness of one’s own position within it, as well as an understanding of the historical and social conditions that led to and continue to shape this position. Central to this is the perspective of viewing one’s own privileges as loss. “Unlearning one’s privilege as one’s loss” (Spivak, 1996, p. 4) entails not simply relinquishing or feeling ashamed of one’s own privileges, but rather examining them within their historical context, questioning and reflecting upon them, and in this sense, not forgetting them but remembering them. In this context, “unlearning ableism” addresses the inquiry and questioning of the aforementioned “internalized expectations of ability and ableist certainties” (Buchner, 2022, p. 204, author’s translation) because they too are part of the violent relations of knowledge and knowledge production, manifesting themselves in educational contexts through form, content, and pedagogical interaction. Self-reflective emotion work could be seen as a facet of learning to unlearn, as it is a part of epistemic change, for “shifting epistemic boundaries is never solely a matter of the cognitive-rational, but always involves aesthetic resources, emotions, and affects” (Brunner, 2020, p. 113, author’s translation).

## 4 Self-reflective emotion work as a systemically oriented group process

Since the 1980s there has been a reflective turn in teacher education, which brought reflexivity to the forefront of discussions about the professionalization of teachers (Haecker, 2022). However, despite the overwhelming emphasis on reflection requirements, they often remain too undifferentiated and abstract in the practice of teacher education, which influences school practice. According to a study by Wyss (2013, as cited in Haecker, 2022), reflections by teachers appear to be “individual, little structured, predominantly purely mental” (Haecker, 2022, p. 100 f., author’s translation). This may be due, in part, to the fact that reflexivity in teacher education is often conveyed as an *individual* strategy and competence—closely linked to the individual-oriented reflection models and tools frequently used in this field and the tendency that “theories of reflexivity are too individualistic and rationalistic” (Burkitt, 2012, p. 464).

At this point, I would like to outline a potential approach for self-reflective emotion work that integrates the aforementioned ideas from the Sociology of Emotions with the process of “unlearning ableism.” The noted proponents of the Sociology of Emotions emphasize the sociality and collectivity of emotions. Social collectives can exhibit various connections depending on the perspective and analytical approach, such as “groups (by way of social category), organizations (by way of formal membership), crowds (by way of physical co- presence), communities (by way of social bonds), or nations (by way of citizenship)” (von Scheve, 2017, n.p.). According to von Scheve (2017), collective emotions are triggered by social identity, social categorization, and the relevance of group concerns, even though they can be experienced situationally by individual subjects. This means that the emotions of individuals and collectives are not viewed individually, but are, as outlined with reference to Ahmed (2004), in a relationship to each other. In the context of Reflexivity, Holmes (2010) views emotions as relationally constructed and emphasizes relationships as central to reflexive practices: “Feelings about and connection to others are crucial to reflexive practices” (Holmes, 2010, p. 143). Shared values, which are also reflected in social norms, now contribute to the fact that individuals “interpret events and situations in similar ways and thus to converge in their emotional reactions” (von Scheve, 2017, n.p.). In the context of ableism, the social collective can be determined through the dominant society, shaped by its structure and culture. The associated collective emotions contribute to the production of social inequalities, “privileging or disprivileging individuals and groups based on the recognition or denial of abilities and legitimizing specific practices of inclusion and exclusion” (Buchner, 2022, p. 203, author’s translation). Teachers—as well as teacher educators—are in most cases part of the dominant society and, due to their specific educational backgrounds as high school graduates and college students, as well as their professional status, they are generally more oriented toward logics of ability and meritocratic principles than other individuals or groups. This description is not intended as an attribution but rather as an attempt to explain ableist practices in schools, which also manifest through the actions of teachers.

In the context of training teachers for inclusive education, which aims to counteract the production of ableist subjects, the exploration of (future) teachers’ own thinking and behavioral patterns, their own concepts of identity, and the embedded emotional patterns should therefore be a core aspect. Building upon the previously outlined aspects of the sociality and collectivity of emotions, the focus here is particularly on self-reflection as a group process that also delves into systemic points of orientation. Because in the relational determination of individual and collective, a systemic principle emerges: contextual orientation, according to which the individual is not viewed in isolation but in the context of their historicity, experiences, social and cultural influences, and social integration.

Accordingly, a systemically oriented group process is designed for participants to experience themselves “much more as social beings than as individual beings” (Mosell, 2016, p. 26), and reflective work is conceptualized as a social practice. Within the framework of applied group dynamics, “situations are created in which the individual can engage with their own experiences and behavior in the group, and from the insights gained in this process, new behavioral possibilities can emerge” (Gilsdorf, 2004, p. 329).

In the context of self-reflective emotion work, this also includes becoming aware of emotions that are closely tied to moral norms and normative expectations, which often unconsciously and pre-reflectively shape the actions of individuals and the group. Additionally, it involves acknowledging as many facets of emotions as possible, which, given the influential nature of feeling rules, is no easy task. However, it is essential if they are to be influenced through reflective engagement. For this purpose, and as designed in applied group dynamics, it is necessary for the individual and the group to be in constant exchange, with the individual’s experiences and reflection processes being relationally linked to the group’s experiences and dynamics (Gilsdorf, 2004). This allows individuals to perceive their own emotional positions and experiences within the context of social relationships, making the social and cultural conditioning of emotions experiential and reflexively accessible. It should be taken into account that the designed reflexive process is itself influenced by emotions, a phenomenon that Burkitt (2012) describes as “emotional reflexivity”: “[…] emotion colours reflexivity and infuses our perception of others, the world around us and our own selves” (Burkitt, 2012, p. 458). This implies a dual perspective for the design of processes in self-reflective emotion work, as reflecting *on* emotions always also involves reflecting *with* emotions.

## 5 Discussion

As has been shown, it is necessary for prospective teachers to engage reflexivity with their own emotions in order to develop a critical understanding of oneself and the social world. In this process, self-reflexive practice itself is shaped by emotions: “Feelings of trust or liking or pleasure, or their opposites, frequently guide reflexive practices” (Holmes, 2010, p. 149). And self-reflexive practice is also shaped by the idea of “what others may be thinking and saying about us and the moral or evaluative stance they may take toward us and our actions” (Burkitt, 2012, p. 469). Two selected aspects are outlined for the discussion, which are to be understood as open questions and topics for discussion regarding the approach of emotion work and its ableism-sensitive implementation and the fact of the emotionalization of reflexivity.

1. Firstly, there is the question of the heterogeneity or homogeneity of the group settings in which (prospective) teachers would work, either with or without individuals with different experiences of marginalization, and what consequences this might have for ableism-sensitive, self-reflective emotion work. The power of feeling rules in a heterogeneous setting could potentially lead to questions about whether the experienced emotions can be allowed. Or the process could be overshadowed by feelings of shame, due to the imagination of value judgments by others, perhaps more so than in homogeneous group settings. Burkitt (2012, p. 462) writes on this: “the uncomfortable emotions that torture us, such as shame, are as much a product of a hyperactive *consciousness* of how others might see us, as of the failure of the unconscious to adequately manage this anxiety.” This could mean that unconscious and pre-reflective emotions and emotional patterns remain concealed and thus evade critical reflection or that the process of unlearning, in Spivak’s sense, is hindered by the feeling of shame (Spivak, 1996; Castro Varela, 2017). However, if teachers, as representatives of the dominant society, work as “equals among equals,” in group processes, there is a risk of reproducing ableist emotional patterns, which in turn undermines the process of “unlearning ableism” and misses the opportunity to “change participants’ relations with others and [to] change how they feel.” (Holmes, 2010, p. 148). Regardless of how we answer the question of group composition, every reflexive process is, as previously mentioned, shaped by emotions (Burkitt, 2012). This requires, in the sense of a reflective cycle, a recurring reflection on the emotions that emerge, and a corresponding methodological response to them.
2. The second question concerns the normative tint that reflection requirements can take on. Critical reflexivity is discussed as a “core element of pedagogical professionalism” (Haecker, 2022). However, the demand for self-reflection also carries the risk of becoming an ableist injunction and practice itself, especially when it becomes established as a norm of reflection. As important as self-reflective competencies are in teacher education for inclusion, they are situated within a professionalization context that aligns with certain concepts of ability and expectations for students. These expectations of ability can be understood as “work on the pedagogical self,” as a call for self-optimization, and thus can also be seen as ableist (Hirschberg, 2016). This not only increases the risk of resistance and refusal of the offer of reflection but also blocks the path for ableism-critical emotion work. Even though resistance is, from a systemic perspective, an essential element of the reflection process, it is important to design the reflection requirements as an open process that incorporates a critical perspective on normative expectations. Haecker (2022) also suggests demystifying and concretizing the so-called reflection competence. In this context, it would be necessary to critically examine what is considered “successful reflection” in the context of ableism-critical emotion work—a question that requires an interdisciplinary, intersectional and process-oriented approach that consistently incorporates the perspective of Disability Studies.

## Data Availability

The original contributions presented in the study are included in the article/supplementary material, further inquiries can be directed to the corresponding author.
